# Elucidation of the Anatomical Mechanism of Nodal Skip Metastasis in Superficial Thoracic Esophageal Squamous Cell Carcinoma

**DOI:** 10.1245/s10434-018-6390-0

**Published:** 2018-02-23

**Authors:** Yuji Kumakura, Takehiko Yokobori, Tomonori Yoshida, Keigo Hara, Makoto Sakai, Makoto Sohda, Tatsuya Miyazaki, Hideaki Yokoo, Tadashi Handa, Tetsunari Oyama, Hiroshi Yorifuji, Hiroyuki Kuwano

**Affiliations:** 1 Department of General Surgical Science, Gunma University Graduate School of Medicine, Maebashi, Gunma Japan; 2 Department of Human Pathology, Gunma University Graduate School of Medicine, Maebashi, Gunma Japan; 3 Department of Diagnostic Pathology, Gunma University Graduate School of Medicine, Maebashi, Gunma Japan; 4 Department of Anatomy, Gunma University Graduate School of Medicine, Maebashi, Gunma Japan

## Abstract

**Background:**

Lymph node metastasis (LNM) is a standard mechanism of cancer progression in esophageal squamous cell carcinoma (ESCC). We aimed to clarify the anatomical mechanism of skip nodal metastasis to mediastinal zones by analyzing the relationship between LNM to sentinel zones and lymphatic vessel counts in the muscle layer adjacent to the outer esophagus.

**Methods:**

We examined the surgical records of 287 patients with ESCC who underwent potentially curative surgery (three-field lymphadenectomy) and whole esophagi, including pharynges and stomachs from 10 cadavers, to determine the number of lymphatic vessels in the intra-outer longitudinal muscle layer adjacent to the outer esophagus of the cervical (Ce), upper thoracic, middle thoracic (Mt), lower thoracic (Lt), and abdominal esophagi (Ae).

**Results:**

The frequency of LNM to the middle mediastinal and supraclavicular zones, including the Mt and Ce, respectively, was lower than to the upper and lower mediastinal and abdominal zone in patients with superficial and advanced thoracic ESCC. In cadavers, the lymphatic vessel counts of the intra-outer longitudinal muscle layer in the Mt and Ce were significantly lower than those of the Lt and Ae, suggesting that lymphatic flow toward the outside of the Mt and Ce was not more abundant than to other sites.

**Conclusion:**

Our anatomical data suggested that the absence of intra-muscle lymphatic vessels in the middle mediastinal and supraclavicular zones causes skip LNM in patients with thoracic ESCC. Thus, standard esophagectomy with lymph node dissection, including distant zones, may be appropriate for treating patients with superficial thoracic ESCC.

**Electronic supplementary material:**

The online version of this article (10.1245/s10434-018-6390-0) contains supplementary material, which is available to authorized users.

The esophagus is a luminal organ with developed longitudinal lymphatic flow.^1^,^2^ It has been suggested that the lymphatic flow networks are associated with the establishment of lymph node metastasis (LNM), which is linked to cancer progression, recurrence, and poor prognoses in patients with esophageal squamous cell carcinoma (ESCC).^2^ Therefore, the fundamental relationships between LNM and lymphatic flow networks should be clarified to control the spread of cancer in patients with ESCC.

It is known that the location of LNM in patients with ESCC depends on the primary tumor site and the depth of tumor invasion. In fact, Yamasaki et al. previously reported important data clarifying the pattern of spread of LNM in patients with cervical ESCC.^3^ Specifically, more than 20% of patients with cervical-centered ESCC at the cervicothoracic junction exhibited LNM to the cervical, supraclavicular, and upper mediastinal zones; however, no patients exhibited LNM in the middle mediastinal, lower mediastinal, and perigastric zones. On the contrary, LNM of thoracic-centered ESCCs at the cervicothoracic junction spread to the cervical, supraclavicular, upper mediastinal, middle mediastinal, lower mediastinal, and perigastric zones. This observation clearly demonstrated the importance of the primary site in the establishment of LMN in cervical ESCC.

In general, LNM in patients with advanced thoracic ESCC spreads to the supraclavicular zone, upper, middle, and lower mediastinal zones, perigastric zone, and celiac zone regardless of the primary tumor site, in contrast to cervical-centered ESCC. Conversely, the establishment of LNM in patients with superficial thoracic ESCC is known to depend on the primary tumor site.^3^ Interestingly, the frequency of LNM to the middle mediastinum in patients with superficial ESCC is lower than to the upper and lower mediastinum, even if the primary site of ESCC is the middle thoracic esophagus (Mt) adjacent to the middle mediastinum.^4^ However, few studies have analyzed the fundamental reasons for this low frequency of LNM to the middle mediastinum, which has been identified as skip nodal metastasis.^5^–^7^ However, the clinical significance of skip nodal metastasis in patients with clinical ESCC remains controversial.^8^–^13^

The purpose of this study was to clarify the mechanism of skip nodal metastasis in patients with superficial thoracic ESCC. Therefore, we examined the anatomical mechanism of skip nodal metastasis to mediastinal zones by analyzing the relationships between LNM to sentinel zones and lymphatic vessel counts in the intra-outer longitudinal muscle layer adjacent to the outer esophagus.

## Materials and Methods

### Clinical Samples

**Fig. 1:**
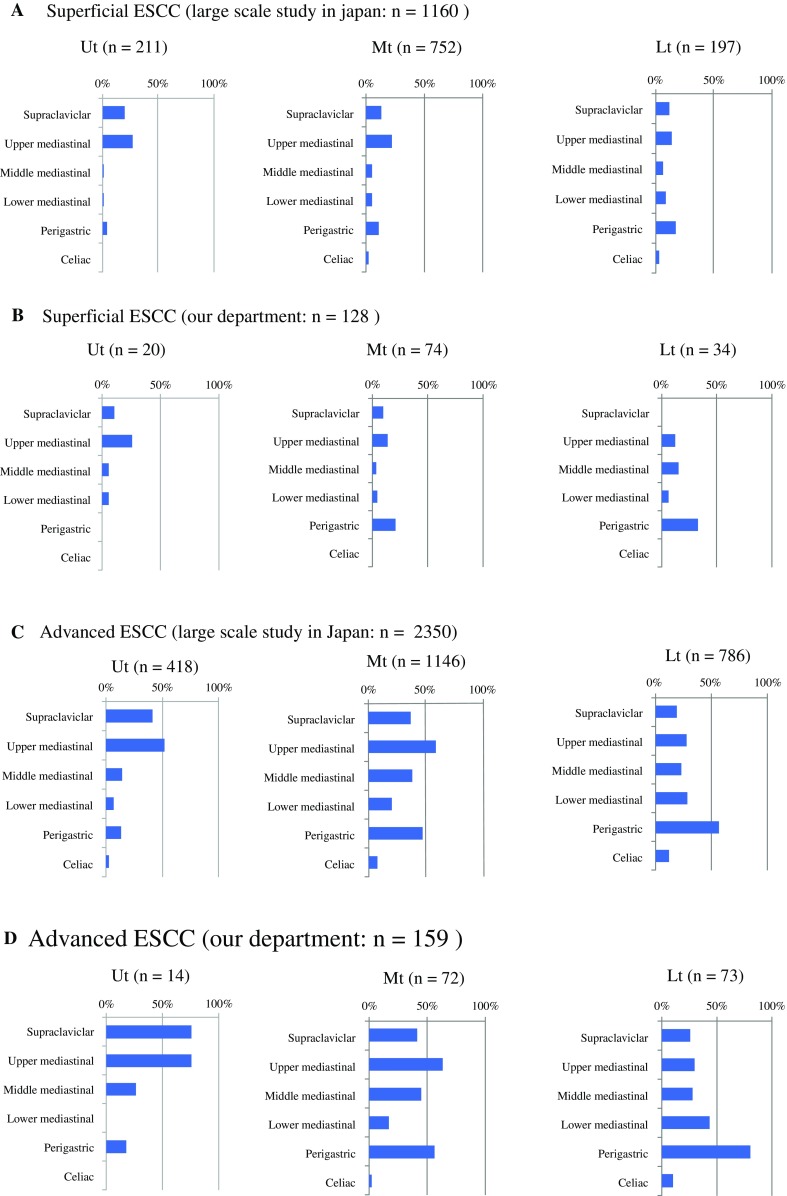
Location and frequency of LNM in patients with ESCC. **a** Location and frequency of LNM for each primary esophageal site, including the Ut (*n* = 211), Mt (*n* = 752), and Lt esophagi (*n* = 197), among patients with T1 superficial ESCC in a prior large-scale study (*n* = 1160)^17^ **b** Location and frequency of LNM for each primary esophageal site, including the Ut (*n* = 20), Mt (*n* = 74), and Lt (*n* = 34), among patients with superficial T1 ESCC in our department (*n* = 128). (**c**) Location and frequency of LNM for each primary esophageal site, including the Ut (*n* = 418), Mt (*n* = 1146), and Lt (*n* = 786), in patients with advanced ESCC in a prior large-scale study (*n* = 2350)^17^ **d** Location and frequency of LNM for each primary esophageal site, including the Ut (*n* = 14), Mt (*n* = 72), and Lt (*n* = 73), among patients with advanced ESCC in our department (*n* = 159). *ESCC* esophageal squamous cell carcinoma, *LNM* lymph node metastasis, *Ut* upper thoracic, *Mt* middle thoracic, *Lt* lower thoracic

**Fig. 2:**
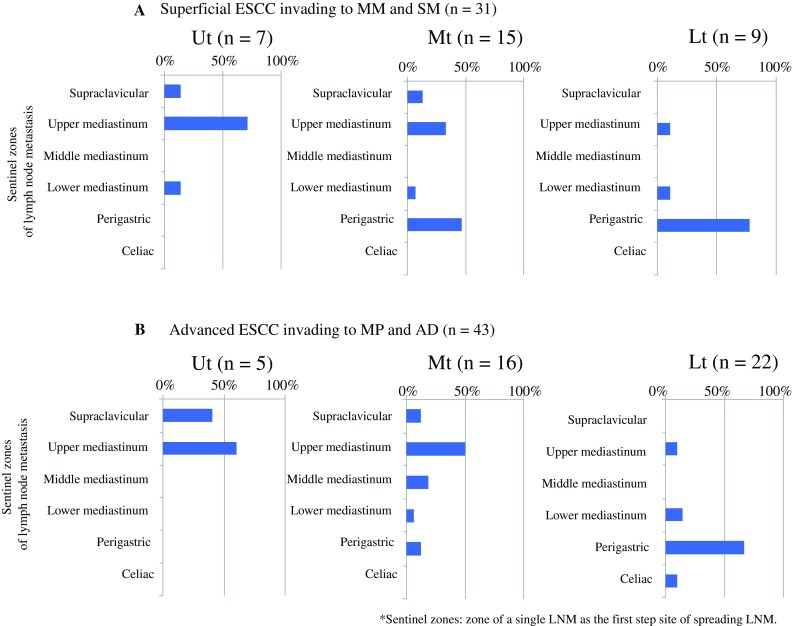
Location and frequency of LNM to sentinel zones in patients with ESCC **a** Location and frequency of LNM to sentinel zones for each primary esophageal site, including the Ut (*n* = 7), Mt (*n* = 15), and Lt esophagi (*n* = 9), among patients with superficial ESCC invading the MM and SM (*n* = 31). **b** Location and frequency of LNM to sentinel zones for each primary esophageal site, including the Ut (*n* = 5), Mt (*n* = 16), and Lt (*n* = 22), among patients with advanced ESCC invading the MP and AD (*n* = 43). The term sentinel zone was used to describe the location of LNM to a single node zone to identify the first step of metastasis. Patients with ESCC and LNM to multiple nodal zones were excluded from this analysis. *LNM* lymph node metastasis, *ESCC* esophageal squamous cell carcinoma, *UT* upper thoracic, *MT* middle thoracic, *Lt* lower thoracic, *MM* muscularis mucosa, *SM* submucosa, *MP* muscularis propria, *AD* adventitia

Surgical specimens were obtained from 287 consecutive patients with ESCC (253 men and 34 women; 128 with superficial ESCC and 159 with advanced ESCC) who underwent surgical resection at Gunma University Hospital, Maebashi, Japan, between 2000 and 2014. All patients underwent esophagectomy with three-field lymphadenectomy and without preoperative adjuvant therapy (electronic supplementary Table 1). The mean patient age was 65.2 years (range 41–86 years). The pathologic characteristics of the specimens were classified based on the 11th edition of the Japanese Classification of Esophageal Cancer,^14^ the Union for International Cancer Control,^15^ and the American Joint Committee on Cancer,^16^ while lymph node zones were classified according to a study by Tachimori et al.^17^ (Figures 1, 2, Table 1). This study has been approved by the Institutional Review Board of Gunma University (approval no. 1561).

**Table 1:** Categorization of node zones, station numbers, and node station names according to the Japan Esophageal Society and American Joint Committee on Cancer

Node zone	Station number (JES)	Name of node station (JES)	Station number (AJCC)	Name of node station (AJCC)
Supraclavicular	101R	Right cervical paraesophageal		(Cervical paraesophageal)
	101L	Left cervical paraesophageal		(Cervical paraesophageal)
	102mR	Right middle deep cervical		
	102 mL	Left middle deep cervical		
	104R	Right supraclavicular	1	Right supraclavicular
	104L	Left supraclavicular	1	Left supraclavicular
Upper mediastinum	105	Upper paraesophageal	3P	Posterior mediastinal
	106pre	Pretracheal	2R	Right upper paratracheal
	106recR	Right recurrent nerve	2R	Right upper paratracheal
	106recL	Left recurrent nerve	2L	Left upper paratracheal
	106tbR	Right tracheobronchial	4R	Right lower paratracheal
	106tbL	Left tracheobronchial	4L	Left lower paratracheal
Middle mediastinum	107	Subcarinal	7	Subcarinal
	108	Middle paraesophageal	8 m	Middle paraesophageal
	109R	Right main bronchus	10R	Right tracheobronchial
	109L	Left main bronchus	10L	Left tracheobronchial
Lower mediastinum	110	Lower paraesophageal	8 l	Lower paraesophageal
	111	Supradiaphragmatic	15	Diaphragmatic
	112	Posterior mediastinum	9	Pulmonary ligament
Perigastric	1	Right cardiac	16	Paracardial
	2	Left cardiac	16	Paracardial
	3	Lesser curvature		
	7	Left gastric artery	17	Left gastric artery
Celiac	8	Common hepatic artery	18	Common hepatic
	9	Celiac	20	Celiac
	11	Splenic artery	19	Splenic
	19	Infradiaphragmatic		
	20	Esophageal hiatus of the diaphragm		

*JES* Japan Esophageal Society, *AJCC* American Joint Committee on Cancer

### Cadaver Samples

Whole, non-cancerous esophagi were obtained from the autopsy specimens of 10 cadavers (four males and six females) without esophageal disease submitted to the Department of Anatomy of Gunma University in 2014. The mean age at the time of death was 86.6 years (range 78–94 years).

### Immunohistochemistry

Immunohistochemical staining was performed using 5-μm-thick sections. All sections were incubated at 60 °C for 60 min, deparaffinized in xylene, rehydrated, and incubated with fresh 0.3% hydrogen peroxide in 100% methanol for 30 min at room temperature to block endogenous peroxidase activity. After the specimens were rehydrated through a graded ethanol series, antigen retrieval was performed in an Immunosaver (Nissin EM, Tokyo, Japan) at 98–100 °C for 60 min, and sections were passively cooled to room temperature. After the sections were rinsed in 0.1 M of phosphate-buffered saline (pH 7.4), non-specific binding sites were blocked via incubation with Protein Block Serum-Free Reagent (Dako, Carpenteria, CA, USA) for 30 min. The sections were then incubated with D2-40 antibody (Dako) at a dilution of 1:200 overnight at 4 °C and at room temperature for 30 min. The reactions were visualized using a Histofine Simple Stain MAX-PO (Multi) Kit (Nichirei, Tokyo, Japan) according to the manufacturer’s instructions. The chromogen 3,3′-diaminobenzidine tetrahydrochloride was applied as a 0.02% solution in 50 mM OF ammonium acetate-citrate acid buffer (pH 6.0) containing 0.005% hydrogen peroxide. The sections were lightly counterstained with hematoxylin, and then mounted. Negative controls were incubated without the primary antibody, and no detectable staining was evident.

### Evaluation of Lymphatic Vessels

Esophageal anatomical specimens were divided into eight sections using three marks, namely the esophageal orifice, tracheal bifurcation, and esophagogastric junction, and round slices were made. The esophagi were divided into five sections as follows: the oral side of the first column was designated the cervical esophagus (Ce); the second and third columns were designated the upper thoracic esophagus (Ut); the fourth and fifth columns were designated the chest Mt; the sixth and seventh columns were designated the lower thoracic esophagus (Lt); and the eighth column was designated the abdominal esophagus (Ae). The sections were stained with D2-40, and the lymphatic vessels of the intra-outer longitudinal muscle layer were counted. Immunohistochemical slides were scanned and evaluated by two experienced researchers.

### Statistical Analysis

For continuous variables, the data were expressed as means ± standard deviations. The relationship between lymphatic duct number and esophagus location were analyzed using analysis of variance (ANOVA). When the results of ANOVA were significant, Tukey’s multiple comparison test was used to assess differences in lymphatic duct number among the Ce, Ut, Mt, Lt, and Ae. Statistical analysis was performed using the JMP software package (SAS Institute Inc., Cary, NC, USA).

## Results

### Location and Frequency of Lymph Node Metastasis to the Sentinel Zones

A large-scale study of T1 ESCC in Japan reported that patients with superficial ESCC of the Mt had nodal skip metastasis and a lower frequency of LNM to the middle mediastinum, as mentioned previously (*n* = 1160) (Fig. 1a).^17^ Similar to the large-scale study, we identified a low frequency of LNM to the middle mediastinum in our cohort of patients with T1 ESCC (*n* = 128) (Fig. 1b), classified according to the guidelines of the Union for International Cancer Control,^15^ American Joint Committee on Cancer,^16^ and Japan Esophageal Society.^14^ In addition, our data were in line with those of the large-scale study among patients with advanced ESCC (Figs. 1c and d). These data define areas of the LNM as node zones; however, to our knowledge, no study has examined the frequency of LNM localized in a single node zone.

In this study, we defined the zone of a single LNM as a sentinel zone, which is also identified as the initial site of LNM. Also in this study, patients with ESCC and LNM to multiple nodal zones were excluded because the purpose of this analysis was to clarify the relationship between the first step of LNM and the site of primary ESCC. The sentinel zones of superficial ESCC in the Ut (*n* = 7) and Lt (*n* = 9) contained the upper and lower mediastinal or perigastric zones, including the paraesophageal lymph nodes in superficial ESCC invading the muscularis mucosa (MM) and submucosa (SM) (Fig. 2a). The sentinel zone of superficial ESCC in the Mt (*n* = 15) did not exhibit LNM to the middle mediastinum, including the paraesophageal lymph nodes of the Mt (Fig. 2a). By contrast, advanced ESCC in the Mt (*n* = 16) invading the muscularis propria (MP) and adventitia (AD) exhibited LNM to the sentinel zones, including the middle mediastinum; however, LNM to the sentinel zone of the middle mediastinum was not observed for advanced ESCC in the Ut (*n* = 5) and Lt (*n* = 22) [Fig. 2b]. Frequent sentinel zones of advanced ESCC were supraclavicular and upper mediastinum in Ut, and upper mediastinum and perigastric in Mt and Lt, respectively (Fig. 2b). The location and frequency of LNM in the T2 cases (*n* = 37) were similar to those in the advanced cases (electronic supplementary Fig. 1).

These results suggest that Mt ESCC invasion to the MP and AD caused direct LNM toward the outside instead of via lymphatic flow as observed in superficial ESCC. Therefore, we hypothesized that the Mt has little lymphatic flow toward the outside, thus affecting the frequency of skip nodal metastasis without LNM to the middle mediastinum in patients with superficial ESCC.

### Lymphatic Vessel Counts of the Intra-Outer Muscle Layer in the Esophageal Areas of Cadavers

**Fig. 3:**
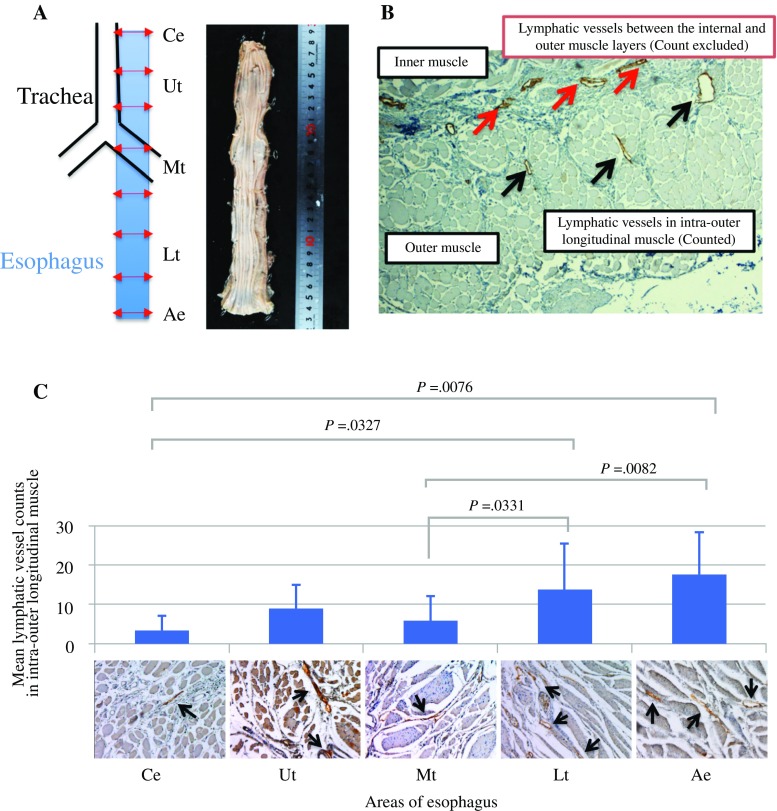
Lymphatic vessel counts of the intra-outer muscle layer in the esophageal areas of 10 cadavers. **a** Location of the evaluated esophageal areas. The esophagi of 10 cadavers were cut into eight slices in the minor axis direction to count the total lymphatic vessels at each esophageal level. **b** Black arrows indicate the counted lymphatic vessels in the intra-outer longitudinal muscle. Red arrows indicate the lymphatic vessels between the internal and outer muscle layers that were excluded from the counts. **c** Relationship between the lymphatic vessel counts of the intra-outer muscle layer in the five esophageal areas. Vessel cells expressing D2-40 were defined as lymphatic vessels. Positive lymphatic vessels of the Ce and Ae were evaluated in one slice from each cadaver, and those of the Ut, Mt, and Lt are presented as the means of two slices. The lymphatic vessel counts in the Mt and Ce were significantly lower than those in the Lt and Ae (Ce: *p* = 0.0327 and *p* = 0.0076, respectively; Mt: *p* = 0.0331 and *p* = 0.0082, respectively). *Ce* cervical esophagus, *Ae* abdominal esophagus, *UT* upper thoracic, *MT* middle thoracic, *Lt* lower thoracic

Yajin et al. previously examined individual differences in lymphatic vessel formation in undefined sites of cadaver esophagi.^18^ However, the previous study method may be unsuitable for evaluating lymphatic flow toward the outside of the esophagus, including areas such as the Ce, Ut, Mt, Lt, and Ae. To overcome this limitation and clarify the anatomical mechanisms of LNM to sentinel zones according to the primary ESCC site, we developed a new method for counting the lymphatic vessels in the intra-outer longitudinal muscle layers adjacent to the outside of the esophageal wall (Fig. 3a). Using this method, we evaluated the lymphatic vessel counts in the intra-outer longitudinal muscle layer alone in 10 cadavers (*black arrows*, Fig. 3b). The average lymphatic vessel counts were calculated in the Ce (3.50 ± 3.74), Ut (9.05 ± 6.04), Mt (6.05 ± 6.21), Lt (13.90 ± 11.70), and Ae (17.67 ± 10.67). The results illustrated that the lymphatic vessel counts in the Mt and Ce were significantly lower than those in the Lt and Ae (Ce: *p* = 0.0327 and *p* = 0.0076, respectively; Mt: *p* = 0.0331 and *p* = 0.0082, respectively) [Fig. 3c], suggesting that lymphatic flow toward the outside of the Mt and Ce was not more abundant than to other sites.

## Discussion

In this study, we clarified the fundamental and anatomical mechanism of LNM to distant sentinel zones, also termed skip nodal metastasis, in superficial thoracic ESCC. The rates of LNM invading the MM and SM were 0–8% and 26–50%, respectively.^19^ Because the anatomical lymphatic network of the SM is abundant in the longitudinal direction, patients with ESCC and SM invasion have a high frequency of LNM to distant mediastinal zones.^1^ Conversely, we demonstrated that lymphatic vessel counts in the intra-outer longitudinal muscle layers were significantly higher in the Ut and Lt than in the Ce and Mt in this study. These results suggested that lymphatic vessel counts in the MP are associated with lymphatic flow from the SM to the outside lymphatic node zone of the esophagus, and this network is associated with skip nodal metastasis of superficial ESCC with SM invasion. Esophageal layers and paraesophageal lymph nodes with abundant lymphatic networks in the mediastinum should be removed to achieve radical resection in patients with both advanced ESCC and superficial thoracic ESCC with SM invasion.

In previous studies, researchers focused on the abundant lymphatic network in the SM layer to explain the establishment of LNM in patients with ESCC; however, the primary focus of our study was to analyze lymphatic vessel counts in the intra-outer longitudinal muscle layer adjacent to the outer esophagus. In fact, we did not observe sentinel zone LNM to the middle mediastinum in patients with superficial thoracic ESCC, and determined that lymphatic vessel counts toward the outside of the Mt in cadavers were lower than those at other esophageal sites. Our report is the first to uncover the fundamental mechanism of the absence of sentinel zone LNM to the middle mediastinum in patients with superficial thoracic ESCC by focusing on differences in lymphatic vessel counts in the Mt. In other words, it was suggested that paraesophageal LNM in advanced Mt ESCC might be caused via lymphatic vessels of intra-outer longitudinal muscle and/or direct invasion, and that paraesophageal LNM via lymphatic vessels, in spite of the superficial Mt ESCC, might associate with poor prognosis such as advanced ESCC.

In this study, we hypothesized that lymphatic vessel counts in the intra-outer longitudinal muscle layer are important for the establishment of skip nodal metastasis in patients with superficial thoracic ESCC. Regarding clinical ESCC samples, Figs. 1 and 2 reveal that the frequency of LNM to the upper mediastinal zones is higher than to the supraclavicular and middle mediastinal zones in both superficial and advanced ESCC. Moreover, we demonstrated via cadaver analysis that lymphatic vessel counts in the intra-outer longitudinal muscle layer in the Ce and Mt, including the supraclavicular and middle mediastinal zones, are lower than in the Ut. These data are consistent with our hypothesis. However, this study was limited by the absence of an evaluation of the clinical significance of lymphatic vessel counts in the intra-outer longitudinal muscle layer using several levels in ESCC samples. Further study is needed to clarify the significance of the lymphatic vessel counts in the muscle layer using clinical ESCC samples with or without LNM.

## Conclusion

LNM skips the middle mediastinal zone in patients with superficial thoracic ESCC due to lower intra-muscle lymphatic vessel counts in the Mt. Our data explain the appropriateness of standard esophagectomy with lymph node dissection, including sentinel node zones, for treating patients with superficial thoracic ESCC, including those with SM invasion.

## Electronic supplementary material

Below is the link to the electronic supplementary material.

Supplementary material 1 (DOCX 65 kb)

Supplementary material 2 (TIFF 7106 kb). Location and frequency of LNM in patients with enrolled T2 ESCC. Location and frequency of LNM for each primary esophageal site, including the Ut (*n* = 1), Mt (*n* = 21), and Lt (*n* = 15), among patients with enrolled advanced T2 ESCC in our department (*n* = 37). *LNM* lymph node metastasis, *ESCC* esophageal squamous cell carcinoma, *UT* upper thoracic, *MT* middle thoracic, *Lt* lower thoracic
