# Supplementary material for: Elucidation of the Anatomical Mechanism of Nodal Skip Metastasis in Superficial Thoracic Esophageal Squamous Cell Carcinoma
Source: Ann Surg Oncol. 2018 Feb 23;25(5):1221–8. doi: 10.1245/s10434-018-6390-0 (PMC5891562; doi:10.1245/s10434-018-6390-0)
Supplement: Supplementary file 1 — Supplementary material 1 (DOCX 65 kb) [file 10434_2018_6390_MOESM1_ESM.docx]

| Supplementary table1. Patients characteristics | | | |  |  |
| --- | --- | --- | --- | --- | --- |
|  |  | Ut (n=34) | Mt (n=146) | Lt (n=107) | p-value |
| Age | Mean(±SD) | 64.6±8.1 | 65.0±8.5 | 65.9±8.0 | 0.5683 |
| Sex | Male | 33 | 127 | 93 | 0.1421 |
|  | Female | 1 | 19 | 14 |  |
| UpT | Tis | 2 | 3 | 1 | **0.0076** |
|  | T1 | 18 | 71 | 33 |  |
|  | T2 | 1 | 21 | 15 |  |
|  | T3 | 10 | 44 | 54 |  |
|  | T4 | 3 | 7 | 4 |  |
| UpN | N0 | 15 | 64 | 42 | 0.974 |
|  | N1 | 8 | 39 | 27 |  |
|  | N2 | 7 | 26 | 24 |  |
|  | N3 | 4 | 17 | 14 |  |
| UpM | M0 | 31 | 132 | 97 | 0.9901 |
|  | M1 | 3 | 14 | 10 |  |
| UpS | S0 | 2 | 3 | 1 | 0.3119 |
|  | S1 | 9 | 53 | 24 |  |
|  | S2 | 9 | 28 | 29 |  |
|  | S3 | 11 | 48 | 42 |  |
|  | S4 | 3 | 14 | 11 |  |
| Lymph node number | | 2.69±4.69 | 2.2±3.98 | 3.32±6.57 | 0.3569 |
| Lymphatic vessels counts (Cadavers n=10) | | 9.05±6.04 | 6.05±6.21 | 13.90±11.70 | **0.0351** |
