# Supplementary figures and images for: Elucidation of the Anatomical Mechanism of Nodal Skip Metastasis in Superficial Thoracic Esophageal Squamous Cell Carcinoma
Source: Ann Surg Oncol. 2018 Feb 23;25(5):1221–8. doi: 10.1245/s10434-018-6390-0 (PMC5891562; doi:10.1245/s10434-018-6390-0)

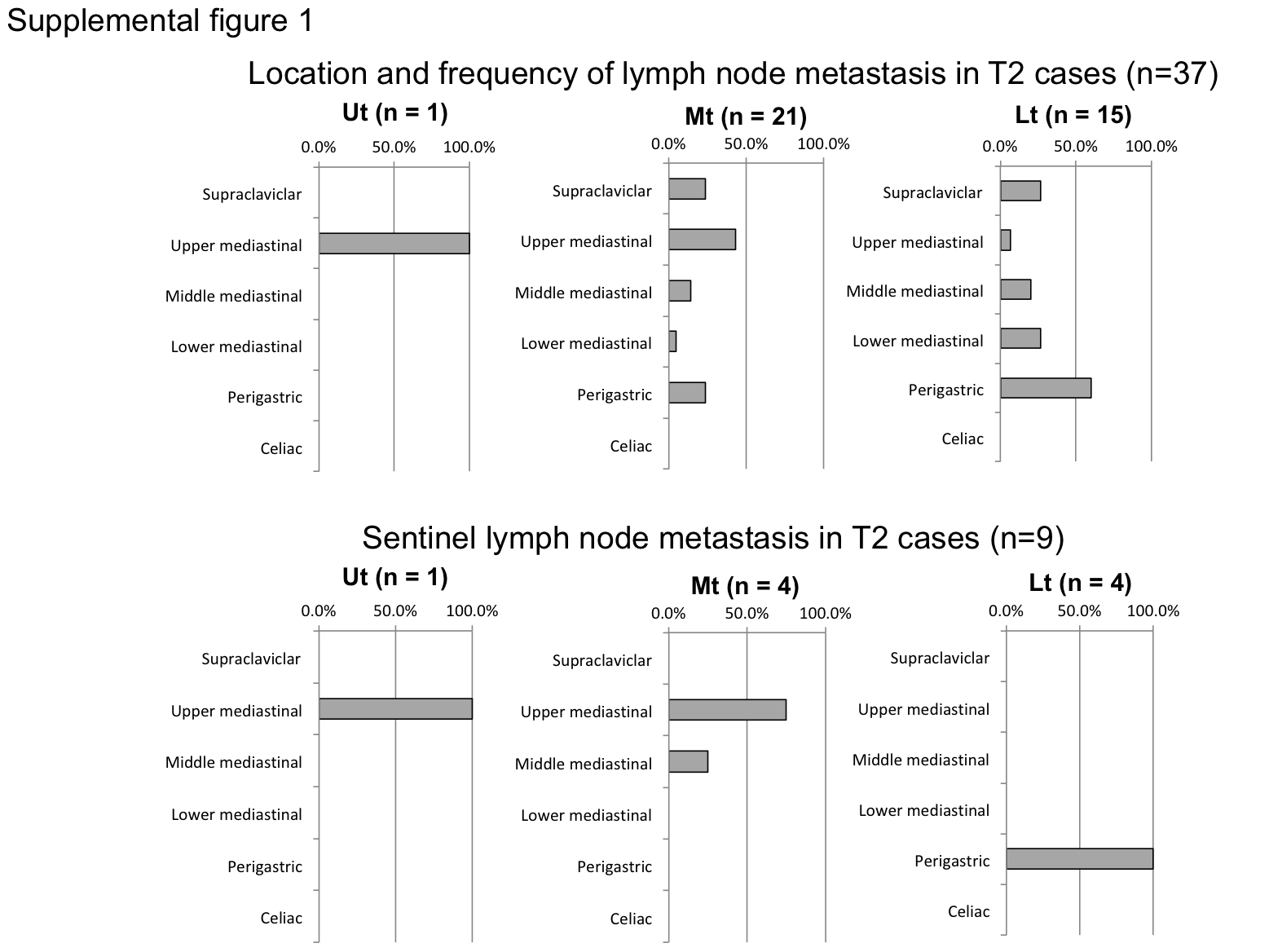

Supplement: Supplementary file 2 — Supplementary material 2 (TIFF 7106 kb). Location and frequency of LNM in patients with enrolled T2 ESCC. Location and frequency of LNM for each primary esophageal site, including the Ut (n = 1), Mt (n = 21), and Lt (n = 15), among patients with enrolled advanced T2 ESCC in our department (n = 37). LNM lymph node metastasis, ESCC esophageal squamous cell carcinoma, UT upper thoracic, MT middle thoracic, Lt lower thoracic [file 10434_2018_6390_MOESM2_ESM.tif]
